# Properties of Particleboard from Oil Palm Biomasses Bonded with Citric Acid and Tapioca Starch

**DOI:** 10.3390/polym13203494

**Published:** 2021-10-12

**Authors:** Radiah Zakaria, Paiman Bawon, Seng Hua Lee, Sabiha Salim, Wei Chen Lum, Syeed Saifulazry Osman Al-Edrus, Zawawi Ibrahim

**Affiliations:** 1 Faculty of Forestry and Environment, Universiti Putra Malaysia, Serdang 43400, Selangor, Malaysia; radiahzakaria@gmail.com (R.Z.); sabiha@upm.edu.my (S.S.); saifulazry@upm.edu.my (S.S.O.A.-E.); 2 Institute of Tropical Forestry and Forest Product (INTROP), Universiti Putra Malaysia, Serdang 43400, Selangor, Malaysia; 3 Institute for Infrastructure Engineering and Sustainable Management (IIESM), Universiti Teknologi MARA, Shah Alam 40450, Selangor, Malaysia; 4 Malaysian Palm Oil Board (MPOB), No. 6, Persiaran Institusi, Bandar Baru Bangi, Kajang 43000, Selangor, Malaysia; zawawi@mpob.gov.my

**Keywords:** citric acid, oil palm trunk, oil palm frond, empty fruit bunch, cyclic aging treatment

## Abstract

The study investigated the effects of the addition of starch on the properties of oil palm biomass particleboard bonded with citric acid. Three kinds of oil palm biomasses were used in this study for the fabrication of particleboard, namely, oil palm frond (OPF), oil palm trunk (OPT), and empty fruit bunch (EFB) particles. Citric acid and tapioca starch at the mixing ratios of 100:0, 87.5:12.5, and 75:25 were prepared at a 60% solid content. A 30% resin content based on the oven-dried weight of the oil palm biomass particles was used. The sprayed particles were pre-dried at 80 °C for 12 h before being hot-pressed at 180 °C and 4 MPa pressure for 10 min. The physical and mechanical properties of the particleboard were evaluated. The mixtures of citric acid and tapioca starch were characterized by thermogravimetric analysis (TGA). Thermal stability of citric acid was reduced after the addition of tapioca starch. The addition of 12.5% tapioca starch improved the bending strength of the particleboard but increased the thickness swelling slightly. All UF-bonded particleboard exhibited significantly inferior performance than that of citric-acid-bonded particleboard. Citric-acid-bonded particleboard maintained its original shape after being subjected to a cyclic-aging treatment, while the UF-bonded particleboard disintegrated half way through the treatment. The performance of EFB particleboard was significantly inferior to its OPT and OPF counterparts.

## 1. Introduction

Malaysia is one of the main palm oil producers and exporters worldwide. As of December 2020, the total oil palm planted area in Malaysia amounted to 5.865 million hectares. Of the planted area, 53.3% is in East Malaysia (Sabah and Sarawak), while the remaining 46.7% is in Peninsular Malaysia [1,2]. According to Khalil et al. [3], the replanting activities and oil palm industry has generated at least 30 million tons of underutilized residues in the form of trunks, fronds, empty fruit bunches, and leaves every year. These oil palm biomasses could be categorized into two general types based on their generation sites. Oil palm trunks (OPT) and oil palm fronds (OPT) are readily available in the planting sites. The combination of OPT and OPF amounted to 75% of the total oil palm biomasses [4]. The remaining 25% are those generated at the mill sites after the extraction of fresh fruit bunches for palm oil, including empty fruit bunches (EFB), palm shell kernels, and palm oil mill effluent (POME). These oil palm biomass residues could be converted into value-added products to avoid wastage. Particleboard is one of the ideal options as any lignocellulosic materials could be used in particleboard production [5]. 

Citric acid is a natural chemical substance that could be found in various fruits and vegetables, especially in citrus fruits. Lemons and limes are among citrus fruits that contain a higher concentration of citric acid [6]. Thanks to its adhesivity and green nature, citric acid could be a good candidate as the main binding agent for wood and wood-based products. Umemura et al. [7] reported that the good adhesivity and bonding properties are a result of the ester linkages formed as the carboxyl groups of citric acid reacts with the hydroxyl groups of the wood constituent. In fact, citric acid has been used as a main binding agent for several types of wood composites such as particleboard, fiberboard, plywood, and laminated veneer lumber [8]. Citric-acid-bonded particleboard is among the most extensively studied wood composites. In recent years, due to the persistent environmental issues, application of non-formaldehyde-based or green binders from renewable resources has become an irresistible general trend. Citric acid could serve ideally to address this problem. 

In the production of particleboard, many additives could be added to the citric acid in order to improve the performance of the resultant boards. For example, Umemura et al. [9] added sucrose to citric acid to improve the bonding performance of the particleboard. On the other hand, teak particleboard was fabricated using citric acid and sucrose, and improvement in dimensional stability was reported [10]. Apart from sucrose, starch could also be added to citric acid to enhance the performance of particleboard. Widyorini et al. [11] fabricated petung bamboo (*Dendrocalamus* sp.) particleboard bonded with different ratios of citric acid and starch. It was found that an addition of 12.5 wt% starch could improve the dimensional stability and bending strength of the particleboard. The authors attributed the improvement to the strong hydrogen bond interactions between citric acid and starch. The authors also reported that the amount of amylose in starch is a deciding factor to the dimensional stability of particleboard. Starch with higher amylose tends to result in particleboard with better dimensional stability as the linear chains of amylose are able to form strong linkages that are responsible for better water resistance. 

A variety of wood and non-wood materials have been used in the production of citric-acid-bonded particleboard such as bamboo [12], teak [10], sweet sorghum bagasse [13], Nipa fronds [14], alang-alang (*Imperata cylindrica*) [15], new giant reed (*Arundo Donax* L.) [16], sugarcane bagasse [17], and rubberwood [18]. However, to the best of the authors’ knowledge, studies on the citric-acid-bonded particleboard made from oil palm biomasses such as oil palm trunk (OPT), oil palm frond (OPF), and empty fruit bunch (EFB) have yet to be reported. The effects of starch addition on the performance of citric acid as a binder for these oil palm biomasses is also unknown. Therefore, the objectives of this study were to investigate the performance of three oil palm biomasses bonded with citric and tapioca starch at different ratios.

## 2. Materials and Methods

### 2.1. Preparation of Materials 

**Figure 1:**
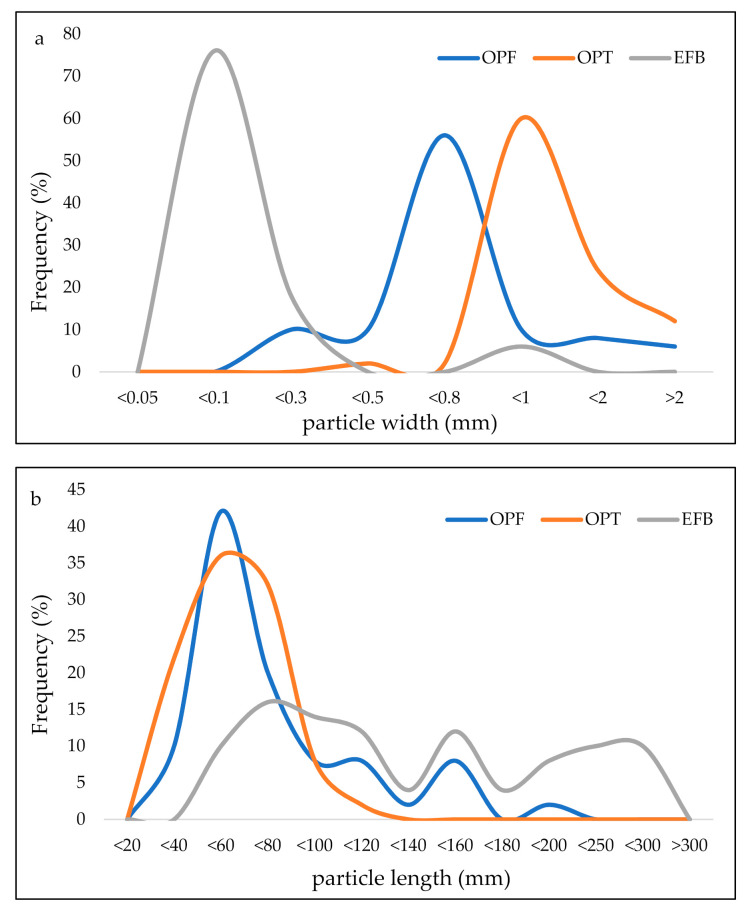
Distribution of width (**a**) and length (**b**) of oil palm frond (OPF), oil palm trunk (OPT), and empty fruit bunch (EFB) particles used in this study.

Three types of oil palm biomasses, namely, oil palm trunk (OPT), oil palm frond (OPF), and empty fruit bunch (EFB) were used in this study. Both OPT and OPF were collected from the felled oil palm trees in an oil palm field at Universiti Putra Malaysia. A portable wood chainsaw (STIHL M170, Waiblingen, Stuttgart, Germany) was used to reduce the size of the OPT and OPF before ground into particle sizes. On the other hand, fibers of EFB were supplied by a local company located at Dengkil, Selangor. The distribution of the particles’ width and length are shown in Figure 1. Most of the OPT and OPF particles had a width ranging between 0.5 to 1.0 mm, while the majority of the EFB particles had a width less than 0.1 mm. In terms of length, the EFB particles used in this study were longer than that of OPT and OPF particles. All of the oil palm biomasses were dried in an oven at 60 °C for 7 days to achieve a 3% moisture content before particleboard fabrication. Citric acid in powder form was purchased from Evergreen Engineering & Resources, Semenyih, Selangor, Malaysia. Tapioca starch was purchased from a grocery store, Serdang, Selangor, Malaysia. Citric acid and tapioca starch were dissolved into distilled water to achieve a 60% resin solid content. The citric acid and tapioca starch mixture ratios were set at 100:0, 87.5:12.5, and 75:25 (*w*/*w*).

### 2.2. Fabrication of Particleboard

**Figure 2:**
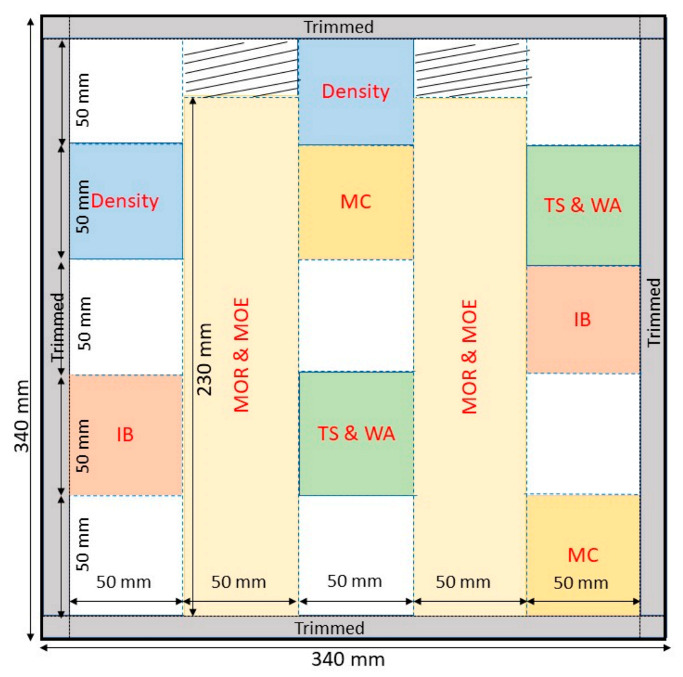
Cutting pattern and sample sampling for evaluation of density, moisture content (MC), thickness swelling (TS), water absorption (WA), internal bond (IB), modulus of rupture (MOR), and modulus of elasticity (MOE).

Single-layer particleboard was produced using citric acid and tapioca starch at different ratios (100:0, 87.5:12.5, and 75:25). Thirty percent resin loading based on the oven-dried weight of oil palm biomass particles were sprayed on the particles. The mixture was then mixed at room temperature for 5 min using a mixer machine (custom made). The mixing process was carried out carefully to ensure the mixture of citric acid and tapioca starch were spread evenly with the oil palm biomass particles. The mixed particle was then oven-dried in an oven at 80 °C for 12 h prior to hot pressing. The oven-dried particles were then hand-formed into a mat using a 340 mm × 340 mm wood mold. The mat was first pre-pressed to compact and then hot-pressed at 180 °C for 10 min with a 4 MPa pressure using a 100 Ton hydraulic laboratory hot press (Carver CMG 100H-15, Ontario, NY, USA). The pressing parameters were chosen according to the study by Lee et al. [8,19], where a pressing temperature of more than 180 °C for 10 min was preferential for citric-acid-bonded particleboard. A pair of 12-mm thick steel bar was used to control the thickness of the board during the hot-pressing process. Particleboards with final dimensions of 340 mm × 340 mm × 12 mm (length × width × thickness) with a target density of 650 kg/m^3^ were produced. Three boards produced from a urea formaldehyde (UF) resin (65% solid content) was used as a control. Ten wt% resin loading was used to produce the control particleboard. One wt% ammonium chloride was added to the UF resin as a hardener. After hot pressing, the boards were conditioned in a conditioning room setting at a temperature of 20 ± 5 °C and a relative humidity of 65 ± 5% until a constant mass was attained. The conditioned particleboards were then trimmed and cut according to the dimension requirements stated in JIS A 5908:2003 [20] for physical and mechanical properties’ evaluation. Figure 2 shows the cutting pattern and sample sampling for the tests. For every particleboard, 2 replicates of samples for each test were obtained. Three replications of particleboard were produced for every citric acid/tapioca starch ratio and UF (control) resin in order to obtain 5 replicates for every physical and mechanical test.

### 2.3. Properties Evaluation

#### 2.3.1. Thermogravimetric Analysis (TGA) of Citric Acid/Tapioca Starch Binder 

TGA of citric acid and topical starch mixture in powdered form was performed under a nitrogen atmosphere using the instrument TGA Q500 V20.13 Build 39 (TA Instruments, Pittsburgh, PA, USA) between 30 and 600 °C (10 °C min^−1^) in the Laboratory of Biocomposite Technology, Institute of Tropical Forestry and Forest Products, Universiti Putra Malaysia, Serdang, Selangor. 

#### 2.3.2. Chemical Composition and Bulk Density of Oil Palm Biomasses 

The determination of chemical content such as lignin, cellulose, hemicellulose, and extractives of the oil palm biomasses were conducted in accordance to the Technical Association of Pulp and Paper Industries (TAPPI) standard. The determination of lignin of the samples was conducted in accordance to TAPPI standard T222 os-74 [21], while the cellulose and hemicellulose was determined according to the procedure specified in TAPPI standard T203 os-74 [22] and Wise et al. [23], respectively. The bulk density of the particles of the oil palm biomasses were determined by using a cylinder method according to the study by Lee et al. [24]. A known weight of oil palm biomasses particles was put into a graduated cylinder half-filled with water. The amount of increment in water volume was recorded, and the bulk density was obtained by dividing the mass of the particles over volume increased. 

#### 2.3.3. Mechanical Properties of Particleboard 

Test specimens were trimmed and prepared according to JIS A 5908:2003 [20] for properties’ evaluation. Mechanical properties including the modulus of rupture (MOR), the modulus of elasticity (MOE), and the internal bond (IB) of the particleboard were evaluated in accordance with JIS A 5908:2003 [20]. The bending properties (MOR and MOE) were evaluated by using a three-point test on samples with dimensions of 230 mm × 50 mm × 12 mm (length × width × thickness). Meanwhile, the IB test was conducted on samples with dimensions 50 mm × 50 mm × 12 mm using a Universal Testing Machine (UTM, Instron-3366, Norwood, MA, USA). Five replicates were tested for each test. 

#### 2.3.4. Physical Properties of Particleboard 

The physical properties of the particleboard including water absorption (WA) and thickness swelling (TS) produced were evaluated in accordance with JIS A 5908:2003 [20]. The samples having a width and length of 50 mm × 50 mm were immersed in water, and the weight and thickness of the immersed were weighed and measured at time intervals of 2 and 24 h. The changes in thickness and weight before and after immersion were expressed as a percentage (%). Cyclic aging treatment was performed according to Kusumah et al. [25]. The immersed samples (after 24 h) were subjected to cyclic aging treatment in the following sequence:

- Drying at 105 °C for 10 h
- Immersion in water at 70 °C for 10 h
- Drying at 105 °C for 10 h
- Immersion in boiling water for 4 h
- Drying at 105 °C for 10 h

The weight and thickness changes of the samples after every stage of the treatment were recorded. Five replicates were used for every test. 

#### 2.3.5. Statistical Analysis 

The data collected were analyzed using Statistical Analysis System (SAS) software (SAS 9.4 solutions, Armonk, NY, United States). Analysis of variance (ANOVA) at a 95% confidence level (*p* ≤ 0.05) was performed. Tukey’s honest significant difference (HSD) test was used to further determine the significant level of average values for each treatment. 

## 3. Results and Discussion 

### 3.1. Chemical Composition and Bulk Density 

The chemical composition and bulk density of the oil palm biomass particles used in this study are shown in Table 1. OPT had the highest lignin content of 26.47% compared with OPF (19.93%) and EFB (21.17%). Meanwhile, EFB had higher cellulose than that of OPT and OPF, while OPF had the highest content of hemicellulose (35.32%). The extractive content for these three oil palm biomasses ranged from 1.54 to 4.80%. The values reported are in agreement with the values reported in Abdul Khalil et al. [26]. They had a high cellulose content, making these oil palm biomasses very suitable in fabricating polymer composites [27]. 

As for bulk density, EFB had the highest bulk density of 670.20 kg/m^3^, while the bulk densities for OPF and OPT were 560.80 kg/m^3^ and 460.00 kg/m^3^, respectively. The findings were comparable to the study by Abdul Khalil et al. [26] who reported that the bulk density of OPF, OPT, and EFB ranged between 600–1200 kg/m^3^, 500–1100 kg/m^3^, and 700–1550 kg/m^3^, respectively. However, the bulk density of oil palm biomasses reported in this study was higher than other agricultural plants such as sweet sorghum bagasse (125 kg/m^3^) [25], kenaf core (118 kg/m^3^) [28], and bamboo (200 kg/m^3^) [29].

### 3.2. Thermogravimetric Analysis (TGA)

**Figure 3:**
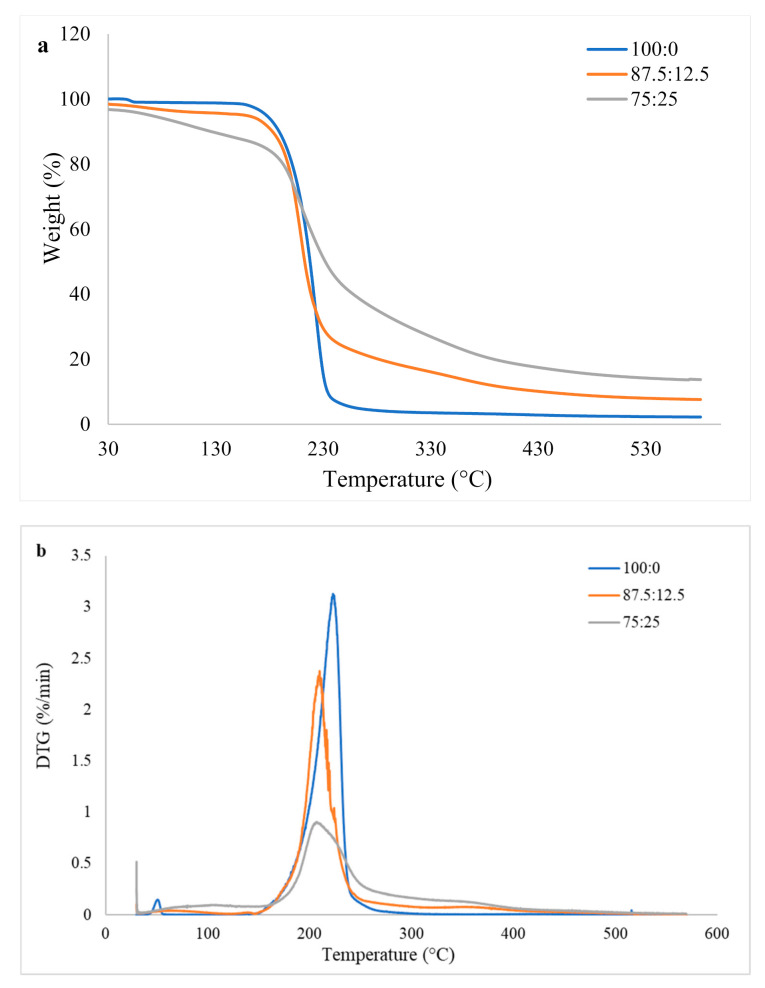
(**a**) Thermogravimetric (TG) and (**b**) derivative thermogravimetric (DTG) curves of binder mixed at different ratios of citric acid and tapioca starch.

Figure 3 displays the thermogravimetric (TG) and derivative thermogravimetric (DTG) curves of binder mixed at different ratios of citric acid and tapioca starch. It can be seen from Figure 3 that pure citric acid started to degrade at around 150 °C and peaked at 224 °C (Figure 3b). The observation is very close to the study by Silva et al. [30] who reported that the decomposition of citric acid occurred between a temperature range of 150 to 220 °C, and the DTG peak was at 210 °C. The authors stated that 95% of the transformation of citric acid occurred within this temperature range. Nevertheless, the addition of starch reduced the initial degradation temperature of the mixture of citric acid and tapioca starch, as seen in Figure 3a. From the DTG curves shown in Figure 3b, one can see that the decomposition temperature was reduced to 210 °C when 12.5% and 25% tapioca starch was added. The observation indicates a lower thermal stability after the addition of starch [31]. A study by Ali et al. [32] revealed that the tapioca starch started to degrade at about 62 °C under a nitrogen atmosphere and continued to lose weight until 500 °C. Therefore, it might be one of the reasons that causes a reduction in the thermal stability of citric acid after the addition of starch. 

The particleboard was hot-pressed at 180 °C in this study. At this temperature, pure citric acid experienced a weight loss of 5.9%, while the mixture of citric acid and tapioca starch at 87.5:12.5 and 75:25 recorded weight losses of 9.0% and 15.8%, respectively. The event implies a lower thermal stability of the mixture at a lower temperature. However, the mixture of citric acid and tapioca starch showed a higher resistance at a higher temperature, and their final residue was much higher than that of pure citric acid. Overall, the mixture of citric acid and tapioca starch at a ratio of 75:25 had the highest T_80_ (temperature for 80% weight loss), which was 388.3 °C, followed by 87.5:12.5 (281.4 °C) and pure citric acid (228.0 °C). The final residues were 13.7%, 7.6%, and 2.2%, respectively. This might be due to the crosslinking of citric acid with starch through covalent bonding. As a higher degree of crosslinking with citric acid was achieved at higher starch loadings, the final residue also increased correspondingly [31]. Additionally, a higher residue could be a result of carbonized material and the ash of starch at a high temperature [32]. 

### 3.3. Mechanical Properties of the Particleboard 

Table 2 demonstrates the mechanical properties of the particleboard produced in this study. 

UF-bonded OPF particleboard had an MOR and an MOE of 6.89 N/mm^2^ and 972.75 N/mm^2^, respectively. The MOR and MOE values increased as citric acid was used as a binder. It is interesting to note that the MOR of the OPF particleboard increased when higher starch was added to the citric acid. As for the MOE, the addition of 12.5% starch increased the MOE of the particleboard, but it started to decrease when 25% starch was added. On the other hand, OPT particleboard experienced the same trend as the OPF particleboard in terms of MOE. However, OPT particleboard showed a decreasing MOR trend as the starch addition increased. The MOR of OPT particleboard bonded with pure citric acid and 87.5CA:12.5 starch did not differ significantly. UF-bonded EFB particleboard had the lowest MOR value of 4.49 N/mm^2^. However, EFB bonded with citric acid exhibited a higher MOR, particularly those bonded with 87.5CA:12.5 starch, which has a statistically higher MOR value than UF-bonded EFB particleboard. The MOE values of the citric-acid-bonded EFB particleboard also displayed a decreasing trend with increasing starch addition, but no significant different was detected between particleboard bonded with pure citric acid and 87.5CA:12.5 starch. 

Citric-acid-bonded particleboard had a higher IB strength than that of UF-bonded particleboard. A very consistent trend was noticeable for the IB strength of citric-acid-bonded particleboard as the IB values decreased with increasing starch addition. The maximum IB strength of pure citric-acid-bonded OPF, OPT, and EFB particleboard were 0.52 N/mm^2^, 0.44 N/mm^2^, and 0.35 N/mm^2^, respectively. However, with the addition of 12.5% starch, the IB strength decreased around 11.5%, 10%, and 28.6% for OPF, OPT, and EFB particleboard, respectively. The IB strength of the particleboard experienced further decrement when 25% starch was added. All the particleboard bonded with citric acid achieved the minimum requirement of IB strength (0.15 N/mm^2^) for type 8 particleboard as specified in JIS A 5908. Overall, with the exception of some outliers, the addition of 12.5% starch was beneficial to the bending strength of the particleboard. Further starch addition (25%) could cause a disastrous effect to the mechanical strength of the fabricated particleboard.

Among the oil palm biomasses, particleboard made from OPF particles seemed to have the highest mechanical properties. This may be due to fact that OPF possesses thick fiber walls. Better bonding could be achieved as the lumen to cell wall ratio is high in OPF, which means OPF particles could be easily compressed closely together [33]. Both OPF and OPT particleboard have significantly better mechanical properties than that of EFB particleboard. One of the probable reasons leading to this observation might be the bulk density of the material itself. Boruszewski et al. [34] reported in their study that the particleboard manufactured from poplar with a lower bulk density achieved a more compacted structure. As a result, minimum voids existed in the boards and correspondingly lead to better mechanical strength. As shown in Table 1, EFB had the highest bulk density followed by OPF and OPT. Lower bulk density inevitably leads to a higher compaction ratio, and the mechanical properties of the boards also increased proportionately with an increasing compaction ratio [24]. In addition, the EFB was reported to have traces of oil on the fiber surfaces [35]. These oil traces caused the EFB to have lower wettability, which prevents the adhesive to be spread evenly on the surfaces of the particleboard. Consequently, it is very difficult for the EFB particleboard to achieve an effective bonding, and subsequently this leads to poor mechanical strength. 

### 3.4. Physical Properties 

Thickness swelling (TS) and water absorption (WA) of the particleboard fabricated in this study are listed in Table 3.

After 2 h immersion in water, the TS_2h_ of the UF-bonded OPF, OPT, and EFB particleboards were 48.07%, 44.19%, and 90.86%, respectively. Meanwhile, TS_24h_ values were 71.22%, 59.96%, and 144.02%, respectively. The observation is unsurprising as UF resin is known to be very instable at high relative humidity owing to its susceptible aminomethylene linkage [36]. However, the TS values of the particleboard dropped significantly when citric acid was used as a binder. The TS_24h_ values of OPF, OPT, and EFB particleboards bonded with pure citric acid (100:0) were approximately one-sixth, one-fifth, and one-seventh of their respective UF counterparts. Both OPF and EFB particleboard bonded with pure citric acid had significantly lower TS_24h_ values than the particleboard bonded with starch addition. The findings were in agreement with Widyorini et al. [11] who reported that the TS of bamboo particleboard increased along with increasing starch content. The authors attributed the increment of the TS to the solubility of starch in water. However, OPT particleboard bonded with 87.5CA:12.5 starch exhibited the lowest TS_24h_ value of 7.28%. 

Similarly, particleboard bonded with citric acid had a significantly lower WA_2h_ (46.97% to 93.29%) and WA_24h_ (57.25% to 123.02%) than UF-bonded particleboard (102.10% to 176.64% and 128.01% to 185.55%, respectively). The findings indicate that the citric acid inhibited water absorption by the particleboard during the water-immersion test [25]. Among oil palm biomasses, EFB particleboard had higher WA values compared to that of the OPF and the OPT particleboards. The OPT particleboard had the lowest TS and WA values. Hashim et al. [37] stated that the particleboard made from non-trunk particles typically had higher TS or poor dimensional stability. As discussed previously, the surfaces of EFB are hydrophobic due to the existence of oil. This hydrophobic characteristic leads to poor resin spreading and prevents good particle–particle bonding [38]. Therefore, EFB particleboard has a very low dimensional stability. 

**Figure 4:**
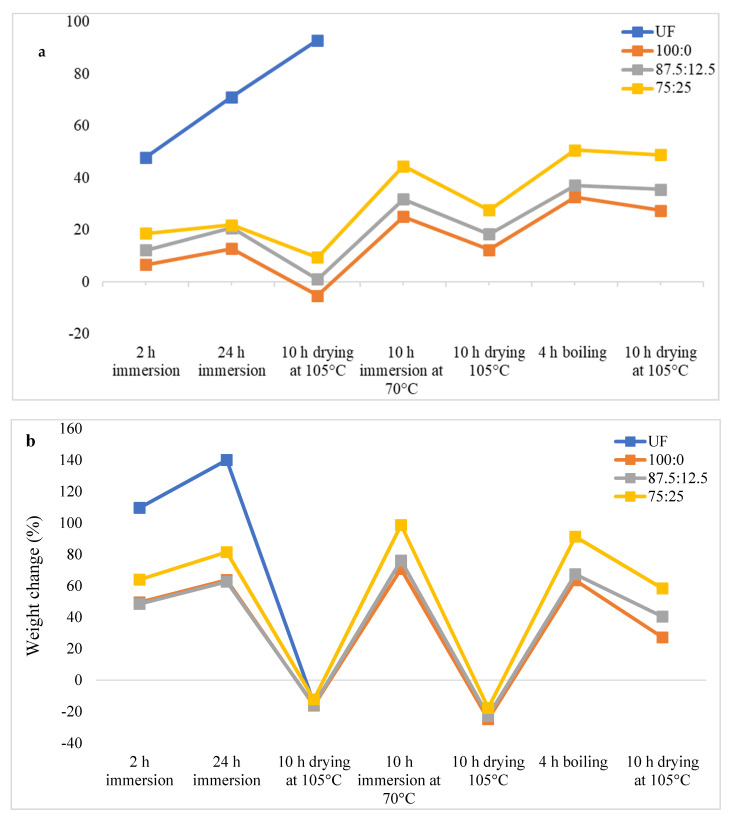
Thickness change (**a**) and weight change (**b**) of oil palm frond particleboard during cyclic-aging treatment.

**Figure 5:**
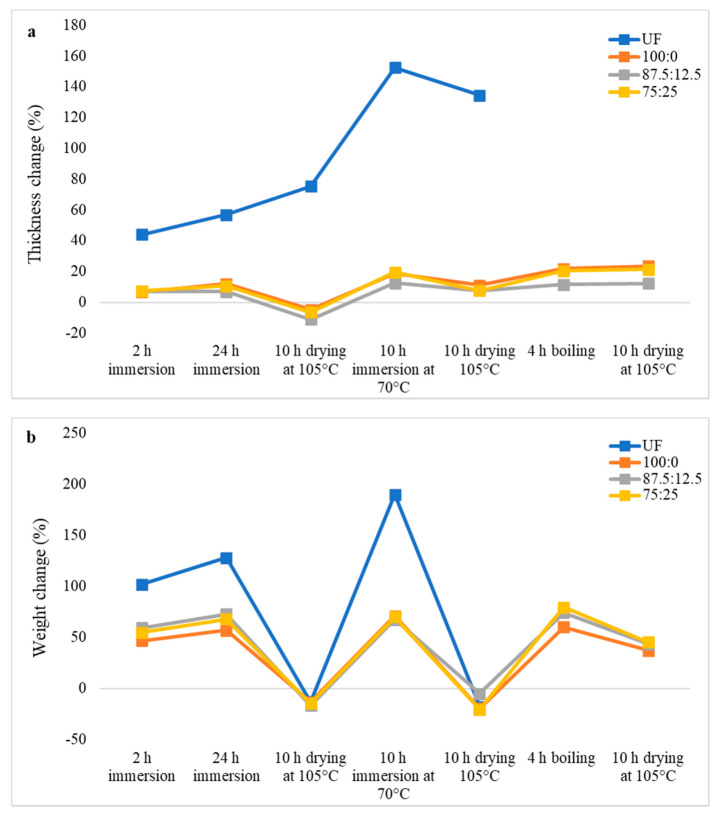
Thickness change (**a**) and weight change (**b**) of oil palm trunk particleboard during cyclic-aging treatment.

**Figure 6:**
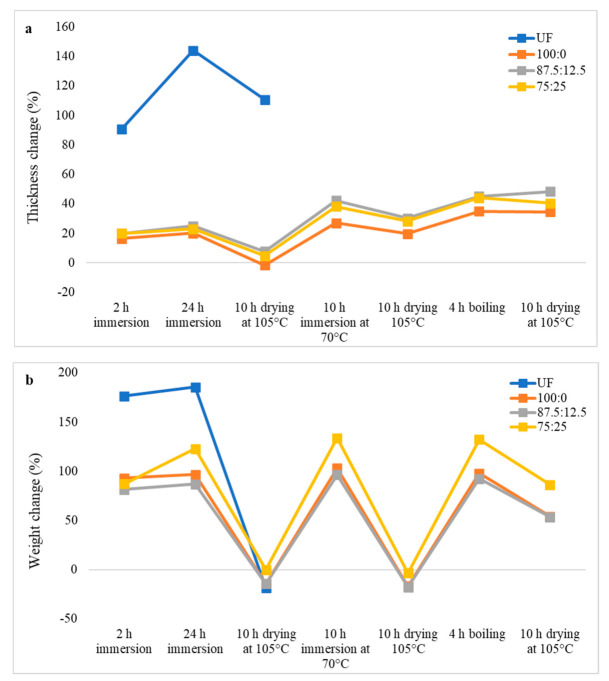
Thickness change (**a**) and weight change (**b**) of empty fruit bunch particleboard during cyclic-aging treatment.

The thickness and weight changes of oil palm biomass particleboards during cyclic aging treatment are shown in Figure 4, Figure 5 and Figure 6.

As shown in Figure 4, UF-bonded OPF particleboard was disintegrated after 24 h of immersion in water followed by drying at 105 °C for 10 h, which indicates very poor dimensional stability. In comparison, OPF particleboard bonded with citric acid displayed a much better dimensional stability compared to that of the UF-bonded particleboard. After boiling in hot water for 4 h, the thickness changes of the particleboard bonded with pure citric acid (100:0) was 32.82%. Meanwhile, particleboard bonded with 87.5CA:12.5 starch and 75CA:25 starch recorded thickness-change values of 37.18% and 50.77%, respectively. The trend of weight change (Figure 4b) during and after the cyclic-aging treatment mirrored that of the thickness change as the particleboard bonded with pure citric acid exhibited the lowest weight-change value.

UF-bonded OPT particleboards are the most dimensionally stable ones compared to that of the OPF and EFB particleboards. UF-bonded OPT particleboard experienced disintegration after the second drying stage at 105 °C, right after immersion in warm water at 70 °C for 10 h. Dissimilar to OPF particleboard, OPT particleboard bonded with 87.5CA:12.5 starch displayed the lowest thickness change of 11.69% after being boiled in hot water for 4 h (Figure 5a). The thickness change values of citric-acid-bonded OPT particleboard was more than two-fold lower than its OPF counterparts. Nevertheless, OPT particleboard bonded with pure citric acid exhibited the lowest weight change after 4 h of boiling in hot water (Figure 5b). 

For EFB particleboard, UF-bonded particleboard experienced disintegration after the first drying stage at 105 °C, which was similar to UF-bonded OPF particleboard. The thickness change value of EFB particleboard bonded with pure citric acid was 35.08% after being boiled in hot water for 4 h (Figure 6a). Meanwhile, the weight values of EFB particleboard bonded with citric acid showed a very drastic change during each stage of the treatment (Figure 6b). 

Generally, in most of the cases, the thickness and weight change values increased along with increasing starch content. OPT particleboard was the only exception, where the particleboard bonded with 87.5CA:12.5 starch displayed the lowest thickness change. It can be concluded that the addition of starch contributed to the increment of water-soluble compounds in the particleboards. As a result, the water resistance of the adhesive reduced as the particleboards were subjected to repeated immersion cycles. The water penetrated into the particleboard and weakened the bond of the particleboard [20]. Apart from that, the reduction in thermal stability after the addition of starch might also contribute to this observation. The particleboard was hot-pressed at 180 °C in this study. Based on the TGA curves shown in Figure 3, at this temperature, pure citric acid experienced a weight loss of 5.9%, while the mixture of citric acid and tapioca starch at 87.5:12.5 and 75:25 recorded weight losses of 9.0% and 15.8%, respectively. Therefore, the citric acid compounds that contribute to the adhesiveness became lesser in the particleboard after the addition of starch, especially when 25% of starch was added. 

With the exception of UF-bonded particleboard, all the particleboard bonded with citric acid and the mixture of citric acid and tapioca starch maintained their original shape after cyclic-aging treatment (Table 4). A darker color was observed for the citric-acid-bonded particleboard after the cyclic-aging treatment. On the other hand, UF-bonded particleboard either displayed a huge extent of swelling or complete disintegration after the cyclic-aging treatment. 

## 4. Conclusions

The effects of the addition of starch on the properties of oil palm biomass particleboard bonded with citric acid were investigated in this study. Generally, the addition of starch reduced the thermal stability of citric acid when subjected to lower temperatures. However, the mixture of citric acid and tapioca starch showed higher resistance at higher temperatures as indicated by the higher amount of residues compared to that of pure citric acid. In terms of the physical and mechanical properties of the particleboard produced, an addition of 12.5% starch might be beneficial. All of the citric-acid-bonded particleboard showed significantly better performance than that of UF-bonded particleboard. Some of the particleboard displayed better bending strength when bonded with a mixture of citric acid and tapioca starch at ratio of 87.5:12.5. Although some particleboards showed a decreasing trend after an addition of 12.5% starch, the decrement was, however, insignificant compared to those bonded with pure citric acid. The addition of 25% starch was unfavorable as it resulted in an adverse effect on the mechanical properties of the particleboard. On the other hand, a similar observation was obtained for physical properties of the particleboard. The addition of 12.5% starch slightly increased the TS and WA of the particleboard (although some showed a slight decrement) due to the solubility characteristic of starch in water. The increment was, however, acceptable. When subjected to the cyclic-aging treatment, citric-acid-bonded particleboard displayed superior dimensional stability as they maintained their original shape after the treatment. On the contrary, all the UF-bonded particleboard disintegrated half way through the treatment. Among oil palm biomasses, OPT and OPF exhibited better performance than that of EFB in terms of mechanical strength and dimensional stability. In conclusion, citric acid is a promising binder for particleboard as well as other wood composites. The addition of 12.5% starch could be beneficial to the mechanical properties, but addition beyond that loading is not recommended.

## Figures and Tables

**Table 1:** Chemical composition and bulk density of oil palm frond, oil palm trunk, and empty fruit bunch particles.

Biomasses	Oil Palm Frond	Oil Palm Trunk	Empty Fruit Bunch
Lignin (%)	19.93 ± 0.26	26.47 ± 0.46	21.17 ± 0.88
Holocellulose (%)	75.26 ± 0.21	71.99 ± 0.48	75.49 ± 0.67
Cellulose (%)	39.95 ± 0.23	42.47 ± 0.19	45.30 ± 0.41
Hemicellulose (%)	35.32 ± 0.41	29.52 ± 0.41	30.19 ± 0.38
Extractive (%)	4.80 ± 0.07	1.54 ± 0.01	3.34 ± 0.12
Moisture content (%)	7.41 ± 0.01	5.82 ± 0.01	6.70 ± 0.01
Bulk density (kg/m^3^)	560.80 ± 15.73	460.00 ± 11.91	670.20 ± 22.40

Note: values after “±” are standard deviations.

**Table 2:** Modulus of rupture (MOR), modulus of elasticity (MOE), and internal bonding (IB) of oil palm biomass particleboard bonded with different binders.

Binder	MOR (N/mm^2^)	MOE (N/mm^2^)	IB (N/mm^2^)
*Oil palm frond*			
UF	6.89 ± 1.57 ^d,e,f^	972.75 ± 51.5 ^e^	0.22 ± 0.06 ^d,e^
100CA:0 starch	7.32 ± 0.65 ^c,d,e^	2299.49 ± 250.38 ^a^	0.52 ± 0.13 ^a^
87.5CA:12.5 starch	9.28 ± 0.63 ^a,b^	2471.98 ± 143.67 ^a^	0.46 ± 0.16 ^ab^
75 CA:25 starch	10.24 ± 0.53^a^	2329.84 ± 114.77^a^	0.34 ± 0.07^bcd^
*Oil palm trunk*			
UF	6.44 ± 0.45 ^e,f,g^	578.12 ± 60.48 ^f^	0.25 ± 0.02 ^c,d,e^
100CA:0 starch	8.75 ± 0.93 ^a,b,c^	1711.96 ± 150.82 ^b,c^	0.44 ± 0.02 ^a,b^
87.5CA:12.5 starch	8.24 ± 0.75 ^b,c,d^	1873.79 ± 62.28 ^b^	0.40 ± 0.03 ^a,b^
75 CA:25 starch	5.64 ± 0.52 ^f,g,h^	1500.05 ± 65.13 ^c,d^	0.37 ± 0.04 ^b,c^
*Empty fruit bunch*			
UF	4.49 ± 0.33 ^h^	854.04 ± 63.81 ^e^	0.12 ± 0.01 ^e^
100CA:0 starch	5.08 ± 0.35 ^g,h^	1287.28 ± 93.51 ^d^	0.35 ± 0.02 ^b,c,d^
87.5CA:12.5 starch	7.15 ± 0.92 ^c,d,e,f^	1285.17 ± 50.57 ^d^	0.25 ± 0.03 ^c,d,e^
75 CA:25 starch	4.49 ± 0.40 ^h^	772.51 ± 64.69 ^e,f^	0.23 ± 0.03 ^c,d,e^

Note: values after “±” are standard deviations. Within the same column, mean values followed by the different letters ^a–h^ were significantly different at *p* ≤ 0.05.

**Table 3:** Thickness swelling (TS) and water absorption (WA) of oil palm biomasses particleboard bonded with different binders after 2 h and 24 h immersion in water.

Binder	TS_2h_ (%)	TS_24h_ (%)	WA_2h_ (%)	WA_24h_ (%)
*Oil palm frond*				
UF	48.07 ± 1.15 ^d^	71.22 ± 2.51 ^e^	109.60 ± 5.31 ^h^	140.01 ± 2.85 ^g^
100CA:0 starch	6.73 ± 0.63 ^a^	12.85 ± 1.00 ^b^	49.46 ± 2.40 ^a,b^	63.69 ± 1.24 ^a,b^
87.5CA:12.5 starch	12.27 ± 0.47 ^b^	20.84 ± 1.11 ^c^	48.48 ± 2.81 ^a,b^	62.60 ± 2.87 ^a,b^
75 CA:25 starch	18.77 ± 1.10 ^c^	22.01 ± 1.99 ^c^	63.91 ± 2.15 ^d^	81.46 ± 5.00 ^d^
*Oil palm trunk*				
UF	44.19 ± 3.08 ^d^	56.96 ± 4.12 ^d^	102.10 ± 1.43 ^g^	128.01 ± 4.03 ^f^
100CA:0 starch	6.65 ± 0.51 ^a^	12.11 ± 0.70 ^b^	46.97 ± 1.91 ^a^	57.25 ± 1.15 ^a^
87.5CA:12.5 starch	6.95 ± 0.40 ^a^	7.28 ± 1.00 ^a^	59.56 ± 1.99 ^c,d^	73.01 ± 1.55 ^c^
75 CA:25 starch	7.38 ± 0.84 ^a^	10.60 ± 0.60 ^a,b^	55.00 ± 0.84 ^b,c^	68.01 ± 1.37 ^b,c^
*Empty fruit bunch*				
UF	90.86 ± 5.11 ^e^	144.02 ± 4.55 ^f^	176.64 ± 6.58 ^i^	185.55 ± 4.71 ^h^
100CA:0 starch	16.53 ± 0.44 ^c^	20.11 ± 0.94 ^c^	93.29 ± 3.17 ^f^	96.68 ± 3.07 ^e^
87.5CA:12.5 starch	20.04 ± 1.34 ^c^	24.63 ± 0.78 ^c^	81.79 ± 2.89 ^e^	87.24 ± 3.63 ^d^
75 CA:25 starch	19.96 ± 1.07 ^c^	23.01 ± 1.27 ^c^	87.26 ± 2.45 ^e,f^	123.02 ± 6.09 ^f^

Note: values after “±” are standard deviations. Within the same column, mean values followed by the different letters, ^a–h^ were significantly different at *p* ≤ 0.05.

**Table 4:** Visual change of oil palm biomass particleboard before and after being subjected to cyclic-aging treatment.

Binder	Before	After
*Oil palm frond*		
UF	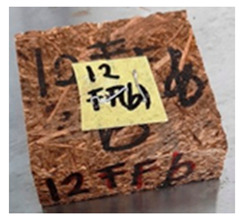	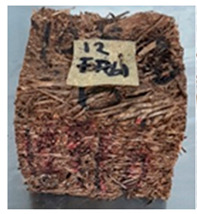 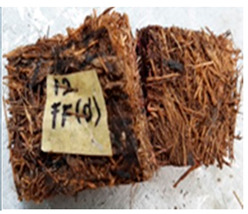
100CA:0 starch	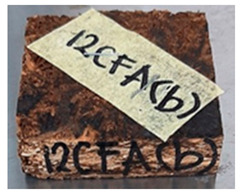	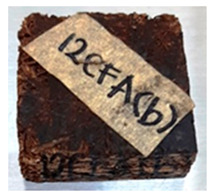
87.5CA:12.5 starch	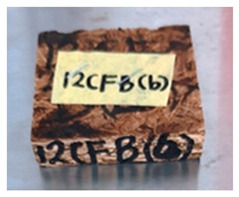	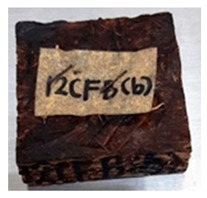
75CA:25 starch	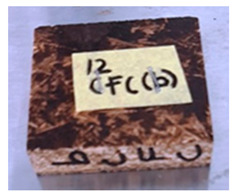	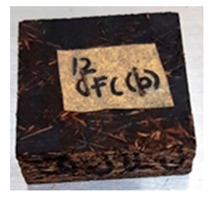
*Oil palm trunk*		
UF	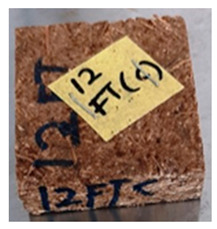	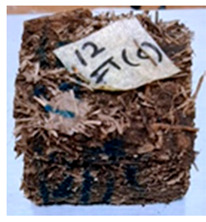 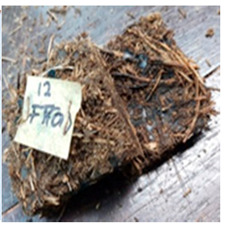
100CA:0 starch	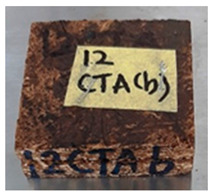	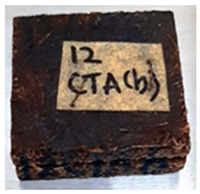
87.5CA:12.5 starch	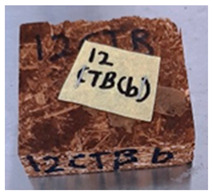	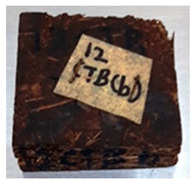
75CA:25 starch	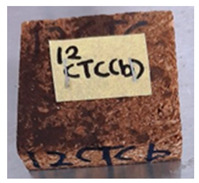	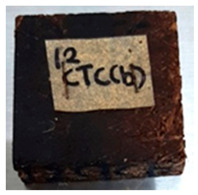
*Empty fruit bunch*		
UF	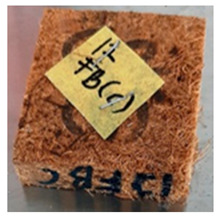	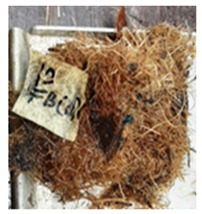 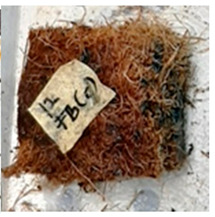
100CA:0 starch	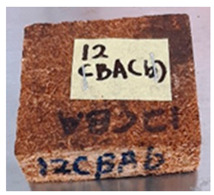	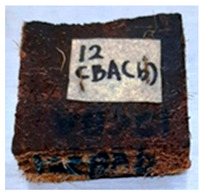
87.5CA:12.5 starch	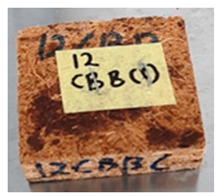	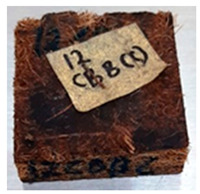
75CA:25 starch	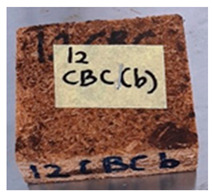	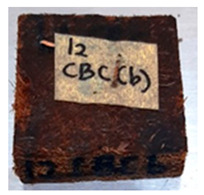

## Data Availability

Not applicable.
